# The hepatoprotective activities of *Kalimeris indica* ethanol extract against liver injury in vivo

**DOI:** 10.1002/fsn3.1241

**Published:** 2019-10-18

**Authors:** Guo‐Kai Wang, Nan Zhang, Yi Wang, Jin‐Song Liu, Gang Wang, Zhong‐Yu Zhou, Chi‐Cheng Lu, Jai‑Sing Yang

**Affiliations:** ^1^ School of Pharmacy Anhui Key Laboratory of Modern Chinese Materia Medica Anhui University of Chinese Medicine Hefei China; ^2^ Bristol‐Myers Squibb Lawrence NJ USA; ^3^ Key Laboratory of Plant Resources Conservation and Sustainable Utilization South China Botanical Garden Chinese Academy of Sciences Guangzhou China; ^4^ Department of Sport Performance National Taiwan University of Sport Taichung Taiwan; ^5^ Department of Medical Research China Medical University Hospital China Medical University Taichung Taiwan

**Keywords:** hepatoprotective effect, in vivo, *Kalimeris indica*, liver injury

## Abstract

*Kalimeris indica* (L.) Sch. Bip. is a traditional Chinese medicine (TCM) and a portion of food used for cooking in China. It has been demonstrated that an ethanol extract of *K. indica* has an anti‐inflammatory effect by inhibition of nitric oxide (NO) production on murine macrophage RAW264.7 cells after lipopolysaccharide (LPS) induction. In this study, the hepatoprotective effects of the total phenolics of *K. indica* (TPK), the total triterpenes of *K. indica* (TTK), and the total flavones of *K. indica* (TFK) from ethanol extracts of *K. indica* were evaluated in Bacille Calmette–Guerin (BCG)/LPS‐induced liver injury in vivo. The treatments of TPK, TTK, and TFK improved liver injury in mice. Additionally, all treatments significantly not only reduced the hepatic malondialdehyde (MDA) content and hepatic total nitric oxide synthase (tNOS) but also induced the hepatic superoxide dismutase (SOD) and glutathione peroxidase (GSH‐Px) activity. The treatments of TPK and TTK significantly reduced the hepatic inducible nitric oxide synthase (iNOS). The treatments of TPK, TTK, and TFK reduced the serum total bilirubin (T‐Bil), and only TFK treatment reduced the serum alanine aminotransferase (ALT). Our results suggest that TPK, TTK, and TFK from ethanol extracts of *K. indica* might play an essential protective role against BCG/LPS‐induced liver injury in vivo.

## 1 INTRODUCTION

Liver injury is a widespread disease throughout the world and usually results from a viral infection, metabolic disorders, chemistry compound, drugs, and/or alcohol (Asrani, Devarbhavi, Eaton, & Kamath, 2019; Cheng et al., 2016). The hepatocyte injury eventually leads to liver fibrosis and cirrhosis (Kourkoumpetis & Sood, 2019; Osna, Donohue, & Kharbanda, 2017). The pathogenesis of hepatic injury is associated with oxidative stress and inflammatory reaction (de Andrade et al., 2015). The liver injury models are known to study the hepatoprotective effects and the underlying molecular mechanisms of new drugs (Kourkoumpetis & Sood, 2019; Yan, Huo, Yin, & Hu, 2018). The Bacille Calmette–Guerin (BCG)/lipopolysaccharide (LPS) method to establish the hepatic injury model in vivo is first reported in 1981 (Ferluga, 1981). BCG/LPS‐induced liver injury in mice is as immune‐mediated chronic hepatitis to further investigate the hepatoprotective actions (Wang et al., 2004). BCG has been reported to induce mononuclear cell infiltration into liver lobules and granuloma formation (Coash, Forouhar, Wu, & Wu, 2012; Ufimtseva, 2013). LPS can cause acute hepatic injury by release of reactive oxygen species (ROS), nitric oxide (NO), glutathione (GSH), and interleukin (IL)‐6, IL‐1β, interferon (IFN)‐γ, and tumor necrosis factor (TNF)‐α (Barboza, da Silva Maia Bezerra Filho, Silva, Medeiros, & de Sousa, 2018; Pan, Long, Yi, & Zhao, 2018). The previous studies have demonstrated that the contents of alanine aminotransferase (ALT), alkaline phosphatase (ALP), TNF‐α, and INF‐γ in the serum and malondialdehyde (MDA) in liver homogenate were increased in the BCG/LPS‐induced liver injury mice; the activities of superoxide dismutase (SOD) and glutathione peroxidase (GSH‐Px) in liver homogenate were decreased. The serious pathological change in liver occurs in the liver of mice with BCG/LPS‐induced liver injury as compared to normal mice (Jiang et al., 2018; Wang et al., 2004).

*Kalimeris indica* (L.) Sch. Bip., an agricultural product, belongs to the family Asteraceae (Compositae) and is found in the eastern Asian countries of China, Taiwan, Korea, and Japan, and in America (Wang et al., 2015, 2017). *Kalimeris indica* is also named Ma Lan, Ji Er Chang, and Tian Bian Ju (Wang et al., 2017). The plant of *K. indica* has been used in traditional Chinese medicine (TCM) and as food for cooking (Wang et al., 2015, 2010). The major clinical applications of *K. indica* include the treatments of the acute gastric abscess, acute orchitis, blood vomiting, conjunctivitis, cold, diarrhea, and gastric ulcer (Wang et al., 2015, 2010, 2017). Chemical investigations of *K. indica* have been found to exert the sixty compounds or phytochemicals, including flavonoids, triterpenes, phenolics, and polysaccharides. (Wang et al., 2017).

Our previous study has shown the hepatoprotective effects of *K. indica* on carbon tetrachloride (CCl_4_)‐induced acute liver injury in primary cultured hepatocytes (Wang et al., 2010). However, the hepatoprotective effects of the ethanol extracts from *K. indica* on BCG/LPS‐induced liver injury of mice are still unclear. In this study, the underlying hepatoprotective effects were investigated to find the total phenolics of *K. indica* (TPK), the total triterpenes of *K. indica* (TTK), and the total flavones of *K. indica* (TFK) from ethanol extracts of *K. indica* in BCG/LPS‐induced liver injury mice model in vivo.

## 2 MATERIALS AND METHODS

### 2.1 Chemicals and reagents

TNF‐α, INF‐γ, IL‐4, and LPS were purchased from Sigma‐Aldrich (Merck KGaA). BCG vaccine was purchased from Shanghai Ruichu Biotech Co., Ltd.. NO, ALT, AST, total bilirubin (T‐Bil), ALP, MDA, SOD, total nitric oxide synthase (tNOS), inducible nitric oxide synthase (iNOS), and GSH‐Px kits were purchased from the Nanjing Jiancheng Bioengineering Institute. Sodium carboxymethyl cellulose (CMC‐Na) was purchased from Sinopharm Chemical Reagent Co. Ltd.. Bifendate pills were purchased from Wanbang Biopharmaceuticals Co. Ltd.

### 2.2 Plant materials

**Figure 1:**
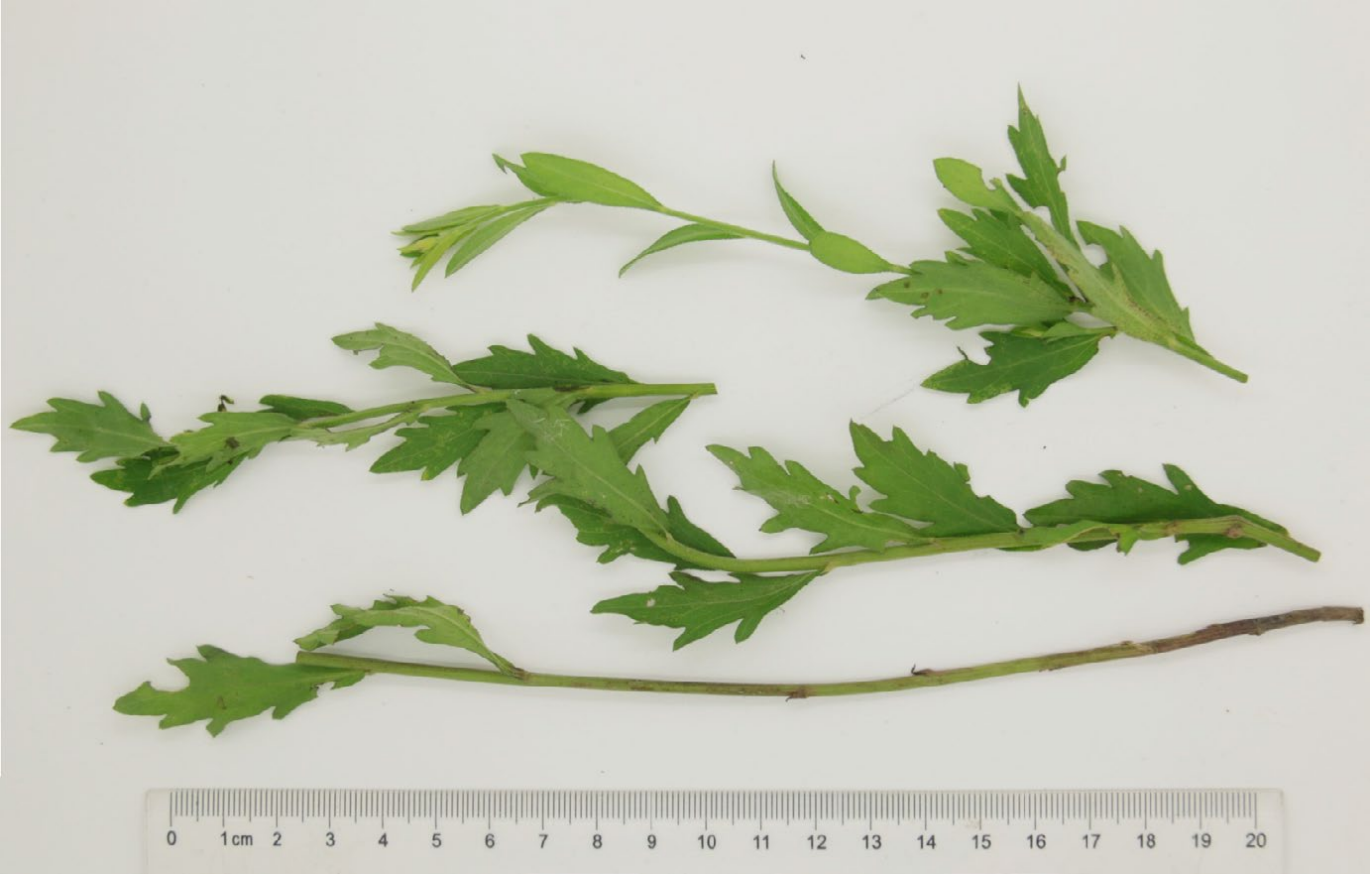
Materia medica of *Kalimeris indica*

The *K. indica* herbs were collected in August 2015 in Hefei, China, and identified by Professor Cheng‐Wu Fang (Anhui University of Chinese Medicine). A voucher specimen (No. KI‐201501) has been deposited in Anhui University of Chinese Medicine. The whole plant of *K. indica* is as shown in Figure 1.

### 2.3 Preparation of crude extracts of *Kalimeris indica*

**Figure 2:**
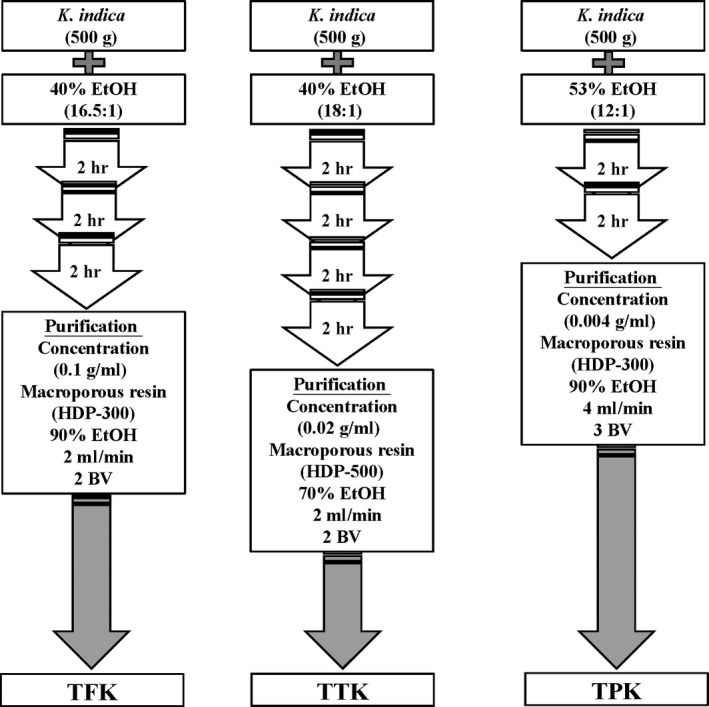
The procedures of preparation of each *Kalimeris indica* extract. *Kalimeris indica* was purified for the total flavones of *K. indica* (TFK), total triterpenes of *K. indica* (TTK), and total phenolics of *K. indica* (TPK), respectively

The air‐dried herbs of *K. indica* (500 g) were treated with 40% ethanol concentration (16.5:1) three times for 2 hr each time. The extracts were evaporated under reduced pressure. The flow rate of purification concentration by macroporous resin (HDP‐300) was 0.1 g/ml, and the sampling mount was 2 ml/min. The concentration and volume of elution ethanol were 90% and 2 bed volume (BV). The total flavones of *K. indica* (TFK) yielded 21.69 g. With rutin as the reference substance, the content of TFK was 56.29%. The air‐dried herbs of *K. indica* (500 g) were crushed and extracted four times with 40% ethanol (1:18) for 1 hr each time. The extracts were evaporated under reduced pressure. The flow rate of purification concentration by macroporous resin (HDP‐500) was 0.02 g/ml, and the sampling mount was 2 ml/min. The concentration and volume of elution ethanol were 70% and 2 BV. The total triterpenes of *K. indica* (TTK) yielded 64.86 g. With oleanolic acid as the reference substance, the content of TTK is 17.72%. The air‐dried herbs of *K. indica* (500 g) were crushed and extracted six times with 53% ethanol l (1:12) two times for 2 hr each time. The extracts were evaporated under reduced pressure. The flow rate of purification concentration by macroporous resin (HDP‐300) was 0.004 g/ml, and sampling mount was 4 ml/min, respectively. The concentration and volume of elution ethanol were 90% and 3 BV. The total phenolics of *K. indica* (TPK) were to yield 55.90 g. With chlorogenic acid as the reference substance, the content of TPK was 24.80%. The crude extracts were dried by freeze‐drying, as previously described (Wang et al., 2015, 2017). The procedures of preparation of each *K. indica* extract can be seen in Figure 2.

### 2.4 Animal experiments and biochemical assays

A total of 120 male Kunming mice (25 ± 5 g) were obtained from the Experimental Animal Center at Anhui Medical University. The animals were treated in accordance with the “Guide for the Care and Use of Laboratory Animals, 8th edition” and approved by the Experimental Animal Ethics Committee of Anhui University of Chinese Medicine. The mice were kept in an environmentally controlled breeding room (temperature 20 ± 2°C, humidity: 60 ± 5%, 12‐hr light/dark cycle) and used for our study when they reached 5 weeks of age. All mice had free access to tap water.

They were randomly divided into 12 groups (ten mice per group). The mice were fed a basal diet for 5 days before being used in this study. All of the mice, apart from those in the mock group (normal mice), were infected by intravenous (I.V.) injection with BCG at 4 mg/0.2 ml/mouse via a tail vein, with the control group getting an equal volume of normal saline. All test samples (TFK, TTK, and TPK) were dissolved in 0.5% CMC‐Na and administered orally every day at a volume of 0.2 ml per mouse (200, 400, and 600 mg/kg, per oral [P.O.]). The bifendate group as a positive control was orally administered every day at a volume of 0.2 ml per mouse (150 mg/kg, P.O.). The mock group received the basal diet without infection and was administered water instead of treatment. The administration of treatment lasted for 10 days, during which the mice were given a normal diet. After the last administration, LPS (10 μg/0.2 ml per mouse) was injected into the tail vein in addition to the mock group to induce liver injury.

**Figure 3:**
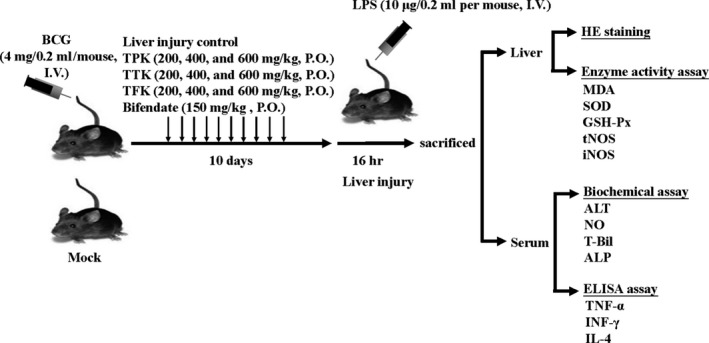
Experimental design of hepatoprotective activities of *Kalimeris indica* in vivo. The mice were infected by intravenously (I.V.) injecting BCG and then orally (P.O.) treated with *K. indica* ethanol extracts. After administration, all mice were sacrificed, and the liver tissue and serum were harvested to further investigate enzyme activity and biochemical assays

After administration, the mice were fasted for 16 hr, and then, blood from the eyeballs and liver was taken. After the blood was left to stand for 45 min, the serum was harvested after centrifugation at 252 *g* for 10 min. The serum levels of ALT, ALP, NO, and T‐Bil were determined according to each kit's instructions. The liver tissue (0.3 g) made of 10% liver homogenate with ice physiological saline, according to the instructions to determine the MDA, SOD, GSH‐Px, tNOS, and iNOS content in the liver homogenates. The levels of TNF‐α, INF‐γ, and IL‐4 in the serum were measured via enzyme‐linked immunosorbent assay (ELISA) according to the manufacturer's kit instructions. After termination of the reaction, absorbance was measured at 450 nm using a microplate reader. All procedures and biochemical methods used in the animal experiments were summarized in Figure 3.

### 2.5 Histological examination

The liver tissue was collected and immediately fixed in 10% formaldehyde. The samples were then dehydrated in graded ethanol (50%–100%) series and cleared in xylene prior to embedding in paraffin. The sections (4–5 μm) were prepared and stained with hematoxylin and eosin (HE) dyes. The areas of necrotic lesions were microscopically evaluated to check the liver injury using a microcomputer image device (MCID) Image analyzer (Imaging Research Inc., St.).

### 2.6 Statistical analysis

The differences between the mean values of the treatment were determined via one‐way analyses of variance (ANOVA) followed by Student's *t* test using SPSS ver. 21.0. software (SPSS, Inc.), and the values were expressed as the mean ± standard deviation (*SD*). The level of significance was uniformly set at *p* < .05.

## 3 RESULTS

### 3.1 Effects of TPK, TTK, and TFK on liver histopathology in mice

**Figure 4:**
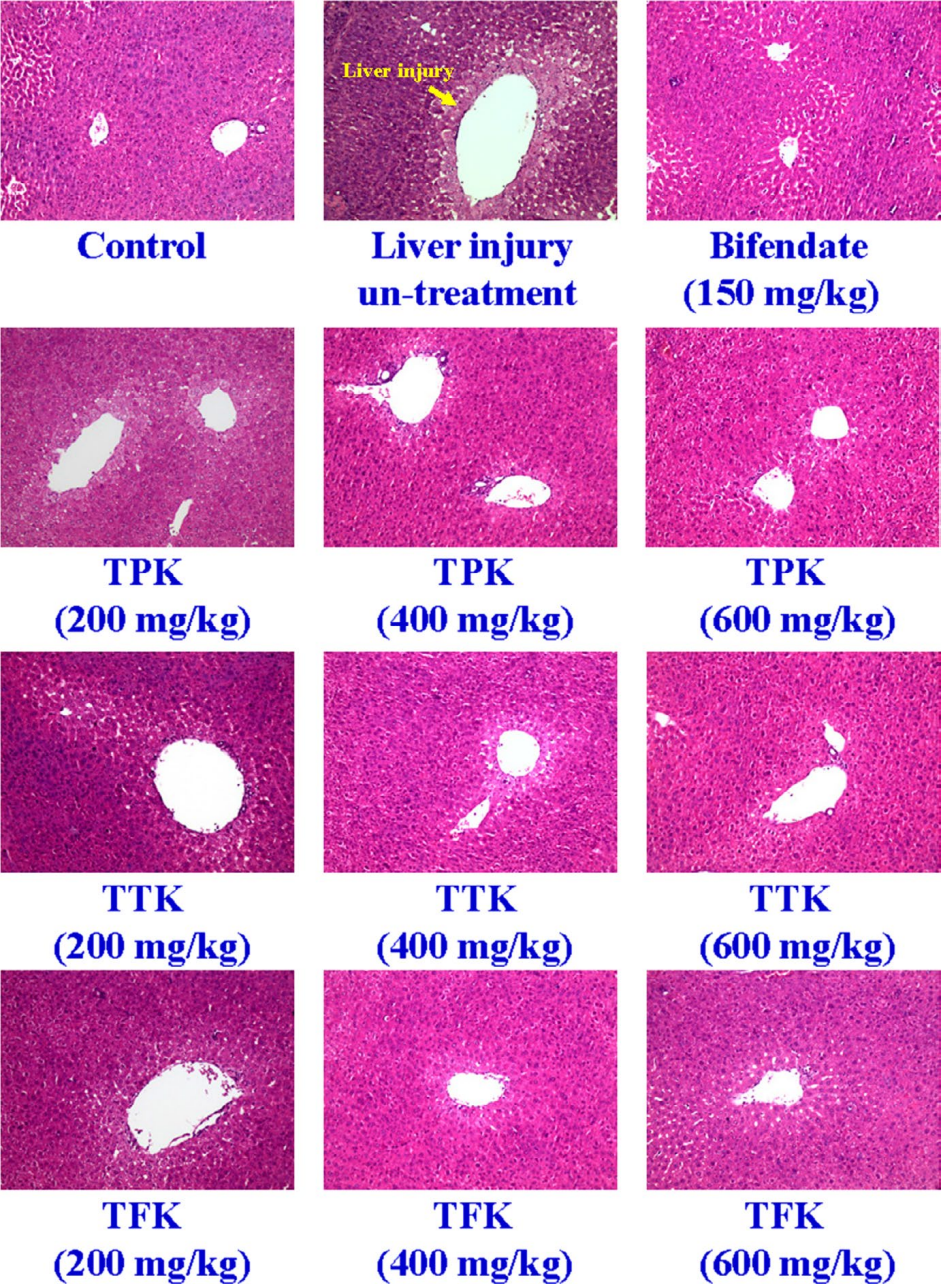
Histological image of liver tissues from mice after each *Kalimeris indica* ethanol extract exposure. All liver sections were subjected to histological study via staining with hematoxylin and eosin (HE). The liver of normal mice showed no histological abnormality. The negative control indicated severe degeneration of hepatocytes with marked mononuclear infiltration (liver injury). The bifendate (150 mg/kg) group was used as a positive control, and the liver injury mice were orally administrated (P.O.) with TPK (200, 400, and 600 mg/kg), TFK (200, 400, and 600 mg/kg), and TTK (200, 400, and 600 mg/kg)

The results of HE staining showed that the liver lobule structure of the normal group was clear. The central cells were the hepatocyte plate, and the cytoplasm was deeply stained. There was no inflammatory cell infiltration in the portal area. The HE staining section of the model group found the central vein around the liver (Figure 4). Liver injury is shown to exert cell swelling and degeneration, partial hepatocyte rupture, massive interstitial hyperemia, large amounts of inflammatory cell infiltration in the portal area, and the surrounding area and large area of flaky necrosis (Kleiner, 2017). The high (600 mg/kg), medium (400 mg/kg), and low dose (200 mg/kg) groups of TPK, TTK and TFK significantly reduced hepatocyte injury and the extent of necrosis (Figure 4). Also, the degree of inflammatory cell infiltration is lighter than the control group (Table 1).

**Table 1:** Effects of TPK, TTK, and TFK on the pathological grading changes in BCG/LPS‐induced liver injury in mice

Groups	Dose (mg/kg)	Degree of liver injury					*p‐*value
		0	I	II	III	IV	
Mock	–	10	0	0	0	0	–
Control (BCG/LPS)	–	0	0	0	0	10	*
Bifendate	150	0	9	1	0	0	#
TPK	200	0	6	2	1	1	#
	400	0	7	2	1	0	#
	600	1	8	1	0	0	#
TTK	200	0	5	2	2	1	#
	400	0	6	2	2	0	#
	600	1	7	1	1	0	#
TFK	200	0	6	2	1	1	#
	400	0	7	1	1	1	#
	600	0	8	1	1	0	#

Note

Each group consists of 10 mice, and the figures represent number of mice per grade.

* *p* < .05, compared with mock (normal) group.

^#^ *p* < .05, compared with control (BCG/LPS) group.

### 3.2 Effects of TPK, TTK, and TFK on MDA, SOD, GSH‐Px, tNOS, and iNOS levels of liver injury in mice

**Figure 5:**
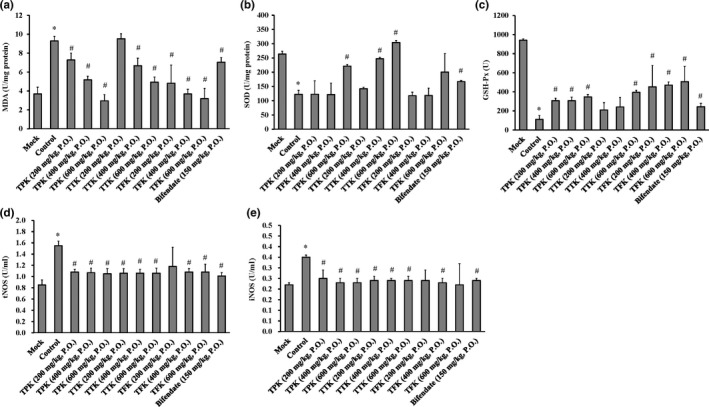
Effects of *Kalimeris indica* ethanol extracts on liver enzyme activity in BCG/LPS‐injured mice. The levels of (a) MDA, (b) SOD, (c) GSH‐Px, (d) tNOS, and (e) iNOS were detected, as described in the MATERIAL AND METHODS section. Data were presented as mean ± *SD* (*n* = 10). *p < .05 compared with mock (normal) group, and ^#^ *p* < .05 compared with control group

The MDA, tNOS, and iNOS levels were significantly increased, and the SOD and GSH‐Px levels were significantly decreased in the liver injury un‐treatment group (control) compared with the mock group (Figure 5). All of the treatment groups saw the MDA level in the liver homogenates effectively reduced (Figure 5a). The TPK at 600 mg/kg and TTK at 400 and 600 mg/kg groups all saw an effective increase in the SOD level (Figure 5b). All the groups effectively increased the GSH‐Px level (Figure 5c) and reduced the tNOS (Figure 5d) and iNOS (Figure 5e) levels when compared to the control group (liver injury mice).

### 3.3 Effects of TFK, TTK, and TPK on ALT, ALP, NO, and T‐Bil levels in the serum of mice

**Figure 6:**
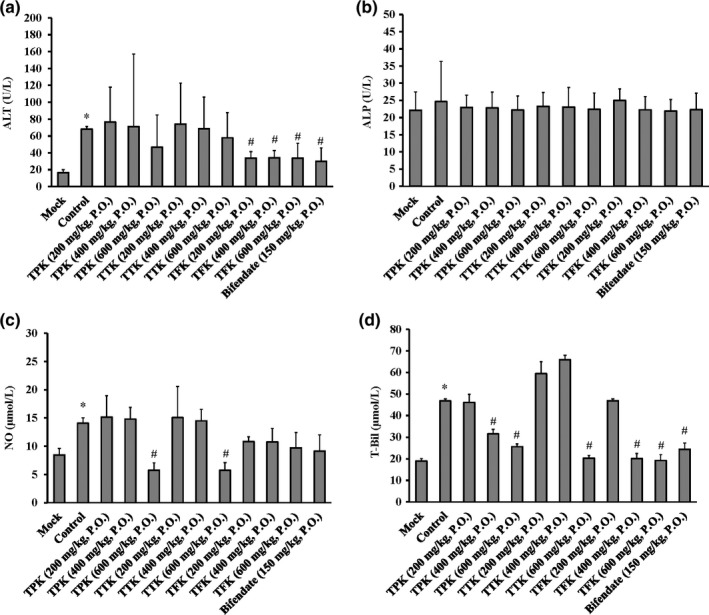
Effects of *Kalimeris indica* ethanol extracts on biochemical analyses of the serum in BCG/LPS‐injured mice. The levels of (a) ALT, (b) ALP, (c) NO, and (d) T‐Bil were determined, as described in the MATERIAL AND METHODS section. Data were presented as mean ± *SD* (*n* = 10). *p < .05 compared with mock (normal) group, and ^#^ *p* < .05 compared with control group

Compared with the mock group, the ALT, NO, and T‐Bil levels of the serum in the liver injury un‐treatment group (control) were significantly increased. Compared with the control group, the TPK and TTK groups were ineffective in the ALT level, and only the TFK (200, 400, and 600 mg/kg) groups significantly reduced the ALT level (Figure 6a). However, there were no significant differences in ALP levels among the all groups treated with or without TFK, TTK, and TPK (Figure 6b). In comparison with control group, the TPK at 600 mg/kg and TTK at 600 mg/kg groups significantly reduced the NO level (Figure 6c). The TPK at 400 and 600 mg/kg groups, the TTK at 600 mg/kg, and the TFK at 400 and 600 mg/kg groups were able to significantly attenuate the high T‐Bil levels (Figure 6d).

### 3.4 Effects of TPK, TTK, and TFK on TNF‐α, INF‐γ, and IL‐4 levels in mice

**Figure 7:**
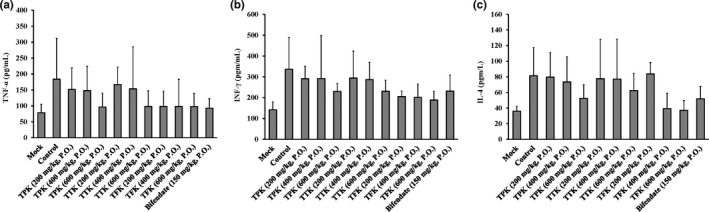
Effects of *Kalimeris indica* ethanol extracts on cytokine activity of the serum in BCG/LPS‐injured mice. The levels of (a) TNF‐α, (b) INF‐γ, and (c) IL‐4 were tested via ELISA, as described in the MATERIAL AND METHODS section. Data were presented as mean ± *SD* (*n* = 10). *p < .05 compared with mock (normal) group

The levels of TNF‐α, IFN‐γ, and IL‐4 in the serum were determined via ELISA. The TNF‐α, IFN‐γ, and IL‐4 levels in the serum in the control group (liver injury un‐treated mice) were slightly increased. The TPK at 600 mg/kg, the TTK at 600 mg/kg, and all of the TFK (200, 400, and 600 mg/kg) groups had the effects on downregulating the TNF‐α (Figure 7a) and INF‐γ (Figure 7b) levels. But only the TPK at 600 mg/kg and the TFK at 400 and 600 mg/kg groups reduced the IL‐4 level (Figure 7c) compared the control group (liver injury mice).

## 4 DISCUSSION

*Kalimeris indica* is a traditional Chinese medicine (TCM) and an agricultural product used for food, especially in China and Taiwan. The whole plant of *K. indica* has been used in the clinical treatments of the acute gastric abscess, acute orchitis, conjunctivitis, diarrhea, gastric ulcer, injuries, and vomiting (Wang et al., 2015, 2010, 2017). The pharmacologic studies showed that the compounds or extracts of *K. indica* have an anti‐oxidant, anti‐inflammatory, antivirus, antimicrobial, antigastric ulcer, and hepatoprotective activities (Wang et al., 2015, 2010, 2017). Our previous study has demonstrated the hepatoprotective effects of *K. indica* in CCl_4_‐induced acute liver injury in vitro (Wang et al., 2010). In the current study, the hepatoprotective action of BCG/LPS‐induced liver injury in vivo was evaluated after individual exposure to TPK, TTK, and TFK from ethanol extracts of *K. indica*. This study is the first to demonstrate that all treatments of *K. indica* ethanol extracts (TPK, TTK, and TFK) have significant hepatoprotective effects on mice with liver injuries. The treatment reduced the hepatic MDA content, hepatic tNOS, and serum T‐Bil, as well as the treatments of TPK and TTK significantly reduced the hepatic iNOS and induced hepatic SOD and GSH‐Px activities.

Numerous agricultural and natural products with anti‐oxidant activity also have the hepatoprotective effect (Al‐Sayed, Abdel‐Daim, & Khattab, 2019; Dai et al., 2018; Lin et al., 2018; Thabet, Youssef, El‐Shazly, El‐Beshbishy, & Singab, 2018). Over fifty compounds, including flavonoids, oils, phenolics, polysaccharides, and triterpenes, were identified from *K. indica* (Wang et al., 2015, 2010, 2017). The biological effects of flavonoids possess anti‐adipogenic, antimycobacterial, hepatoprotective, anti‐inflammatory, immunomodulatory, neuroprotective, anti‐oxidant, hypoglycemic, and anticancer activities (Batra & Sharma, 2013; Chahar, Sharma, Dobhal, & Joshi, 2011; Karker et al., 2016; Wang et al., 2018). The biological effects of triterpenoids exert anti‐allergic, hepatoprotective, anti‐inflammatory, immunomodulatory, anti‐oxidant, hypoglycemic, and anticancer effects (Tenkerian, El‐Sibai, Daher, & Mroueh, 2015). The biological effects of phenolics have antimycobacterial, anti‐inflammatory, immunomodulatory, neuroprotective, anti‐oxidant, hypoglycemic, and anticancer activities (Upadhyay & Dixit, 2015). Our earlier chemical studies on *K. indica* have shown to detect phenolics compounds that include 4‐hydroxyacetophenone, episyringaresinol, epipinoresinol, pinoresinol, vanillin, *p*‐hydroxybenzaldehyde, syringic acid, 3,4‐dihydroxybenzaldehyde, dibutylphthalate, coniferyl alcohol, syringaresinol, lariciresinol, 4‐allyl‐3,5‐dimethoxyphenol, 1‐(3,4,5‐trimethoxyphenyl) ethanone, physcion, chrysophanol, emodin, and physcion (Wang et al., 2015, 2010). Triterpenes compounds were found to include kalimerislactone B, (3β)‐3‐hydroxyolean‐12‐en‐28‐oic acid, soyasapogenol E, 6‐hydroxy‐eudesm‐4 (14)‐ene, 3‐oxo‐dammara‐20 (21),24‐dene, 3β‐acetyl‐dammar‐20 (21),24‐dene, friedelin, lupeone, α‐amyrin, friedel‐3‐ol, olean‐12‐ene‐2β,3β,28α‐triol, gult‐5‐en‐3β‐ol, 3β‐acetyl‐25‐hydroxydammara‐20 (21),23‐diene, and argungenin. The flavonoid compounds contain wogonin, oroxylin A, 7,4’‐dihydroxyisoflavone, apigenin, 7‐hydroxy‐4’‐methoxyisoflavone, rhamnetin, apigenin‐7‐O‐β‐D‐glucoside, and biochanin A (Wang et al., 2015, 2017).

Our results of the histological changes in the liver directly reflected the degree of liver injury and repair in TPK, TTK, and TFK treatment groups from ethanol extracts of *K. indica* (Figure 4 and Table 1). The results showed that the total phenolics fractions (TPK) from ethanol extracts of *K. indica* have the hepatoprotective effects on BCG/LPS‐induced liver injury of mice. TPK significantly reduced the hepatic MDA (Figure 5a), hepatic tNOS (Figure 5c), hepatic iNOS (Figure 5d), and serum T‐Bil (Figure 6d) levels. Additionally, TPK significantly induced the hepatic SOD at 600 mg/kg dosage (Figure 5b) and GSH‐Px (Figure 5c) activities. It has been reported that the phenolics compounds such as hydroxyacetophenone derivatives have anti‐hepatitis B virus infection (Zhao et al., 2015). Pinoresinol (Kim et al., 2010), vanillin (Makni et al., 2011), syringic acid (Itoh et al., 2010), and emodin (Bhadauria et al., 2009) exhibit hepatoprotective effects on CCl_4_‐induced liver injury. On the other hand, emodin has been demonstrated to have a hepatoprotective effect in vitro and in vivo studies that attenuates LPS or acetaminophen‐induced liver injury (Bhadauria, 2010; Ding et al., 2018). Our results suggest that 4‐hydroxyacetophenone, pinoresinol, vanillin, syringic acid, and emodin maybe are the major elements of TPK on hepatoprotective active fractions.

Our results indicated that the TTK significantly reduced the hepatic MDA at 400 and 600 mg/kg dosages (Figure 5a), hepatic tNOS, hepatic iNOS at 400 and 600 mg/kg dosages (Figure 5c and d), and serum T‐Bil at 600 mg/kg dosage (Figure 6d) levels. TTK significantly induced the hepatic SOD at 400 and 600 mg/kg dosages (Figure 5b) and GSH‐Px at 600 mg/kg dosage (Figure 5c). Previous study has shown that the triterpenes compounds, including α‐amyrin (Singh, Arya, Sharma, Dobhal, & Gupta, 2015), have hepatoprotective effects on CCl_4_‐induced liver injury. Our result suggests that α‐amyrin maybe is the major compound of TTK on hepatoprotective active fractions.

Additionally, our finding also demonstrated that the TFK significantly reduced the hepatic MDA at 200, 400, and 600 mg/kg dosages (Figure 5a), hepatic tNOS at 200, 400, and 600 mg/kg dosages (Figure 5c), hepatic iNOS at 400 and 600 mg/kg dosages (Figure 5d), and serum ALT at 200, 400, and 600 mg/kg dosages (Figure 6a), and serum T‐Bil at 400 and 600 mg/kg dosages (Figure 6d) levels. TFK significantly induced the hepatic SOD at 600 mg/kg dosage (Figure 5b) and GSH‐Px at 200, 400 and 600 mg/kg dosages (Figure 5c). It is documented that the flavonoid compounds, including apigenin, exert anti‐oxidative and hepatoprotective effects on paracetamol‐ and *N*‐nitrosodiethylamine (NDEA)‐induced liver injury (Ali, Rahul, Naz, Jyoti, & Siddique, 2014; Raskovic et al., 2017). Biochanin A (Breikaa, Algandaby, El‐Demerdash, & Abdel‐Naim, 2013) and oroxylin A (Zhu et al., 2013) have hepatoprotective effects on CCl_4_‐induced liver injury. Our result suggests that apigenin, biochanin A, and oroxylin A maybe the major bioactive components of TFK on hepatoprotective active fractions.

The hepatic MDA is the major product of lipid peroxidation in liver, and the MDA can reflect the change that is liver damage (Smathers, Galligan, Stewart, & Petersen, 2011). The levels of SOD and GSH‐Px are two important key enzymes in the anti‐oxidative system, and those can reflect the change in the anti‐oxidation effect on the liver (Dunning et al., 2013). Our results demonstrated that the TPK, TTK, and TFK significantly reduced the hepatic MDA (Figure 5a). TPK, TTK, and TFK also significantly induced the hepatic SOD (Figure 5b) and GSH‐Px activities. (Figure 5c). It has been reported that flavonoids, phenolics, and triterpenes have an anti‐oxidation activity by regulating SOD and GSH‐Px activities in liver injuries (Ai, Huang, Liu, Han, & Chen, 2016; Ali et al., 2018; Zhang et al., 2018; Zhao et al., 2012). In a BCG/LPS‐induced liver injury in vivo system, the hepatic tNOS and hepatic iNOS are vital enzymes to regulate the pro‐inflammatory cytokine production or NO signal transduction (Soskic et al., 2011). The level of iNOS was to generate NO by oxidation of L‐arginine to L‐citrulline. Inhibition of tNOS or iNOS enzyme activities protected the liver against BCG/LPS‐induced injury (Abdel‐Salam et al., 2017). Our results showed that TPK, TTK, and TFK significantly reduced the hepatic tNOS or iNOS activities (Figure 5c and d). Flavonoids, phenolics, and triterpenes have hepatoprotective actions by suppressing iNOS expression in liver injury (Ai et al., 2016; Farombi, Shrotriya, & Surh, 2009; Vicente‐Sanchez et al., 2008; Xin et al., 2016). However, the molecular mechanisms of *iNOS* gene regulation in the BCG/LPS liver injury in vitro model indeed need to further study.

**Figure 8:**
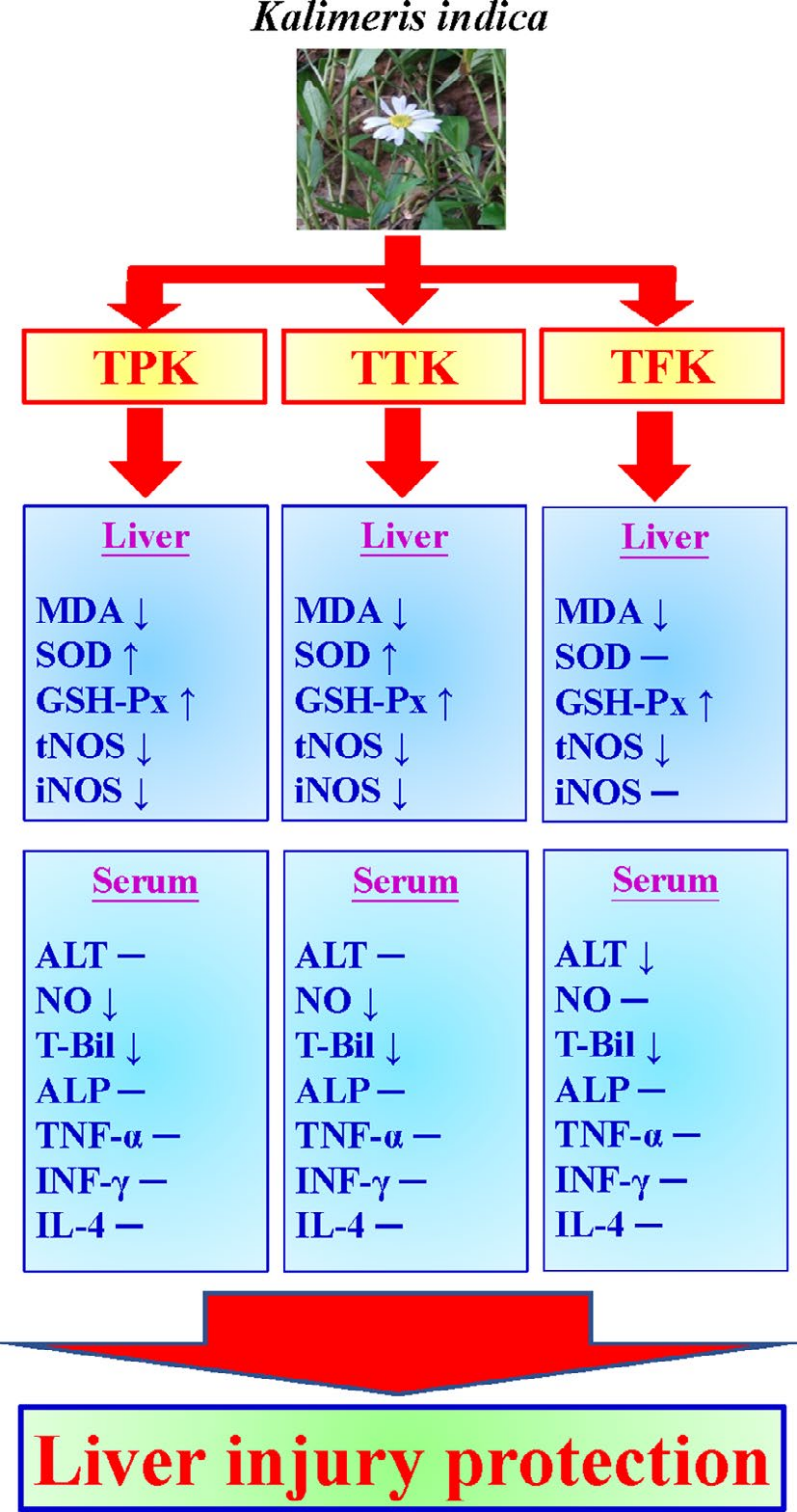
Effects of *Kalimeris indica* ethanol extracts (TPK, TTK, and TFK) on hepatoprotective activities in vivo. *Kalimeris indica* ethanol extracts inhibited enzyme activities (MDA, tNOS, and iNOS) in liver tissues and suppressed T‐Bil and NO levels in the serum from mice against liver injury

In conclusions, our results are in agreement with the measurements of biochemical indicators in the serum and anti‐oxidative indexes in liver homogenate, which confirms that ethanol extracts of *K. indica* possess hepatoprotective action in BCG/LPS‐induced liver injury mice, as illustrated in Figure 8.

## CONFLICT OF INTEREST

The authors declare that they do not have any conflict of interest.

## ETHICAL APPROVAL

This study was approved by the Experimental Animal Ethics Committee of Anhui University of Chinese Medicine.

## INFORMED CONSENT

Written informed consent was obtained from all study participants.
